# Descriptive Anatomy of the Human Teeth

**Published:** 1890-12

**Authors:** 


### Bibliographical.

DESCRIPTIVE ANATOMY OF THE HUMAN TEETH.—By G. V. Black, M. D., D. D. S. Publishers: The Wilmington Dental Manufacturing Company, Philadelphia. Price, $2.50. The author states in his preface that this work is designed to give a knowledge of the details of the specific forms of the human teeth—comprising the various surfaces and surface markings, so that a more complete perception of what compose the individual teeth of man may be acquired. The contents include Dental Anatomy—nomenclature, measurements of the teeth; the description of the various classes of teeth; the permanent and deciduous teeth; the pulp chamber in each class of teeth; arrangement of the teeth; the alveolar process and alveoli; the peridental membrane and gums. Many excellent illustrations assist the reader, and the whole comprises an attractive volume of 153 pages exclusive of the index.

This work, beyond question, furnishes the most elaborate and comprehensive description of the human teeth and dental structures in direct relation with them, which has yet appeared in print, and reflects great credit upon its author, who is widely known for his extensive researches and valuable contributions to dental histology.

